# From Admission to Discharge: Leveraging NLP for Upstream Primary Coding with SNOMED CT

**DOI:** 10.1007/s10916-025-02200-4

**Published:** 2025-05-22

**Authors:** Elisa Asensio Blasco, Xavier Borrat Frigola, Xavier Pastor Duran, Artur Conesa González, Narcís Macià, David Sánchez Barcenilla, Ricardo Garrido Bejar, Santiago Frid

**Affiliations:** 1 Clinical Informatics Service, Hospital Clínic de Barcelona, Barcelona, Spain; 2 Clinical Foundations Department, Universitat de Barcelona, Barcelona, Spain; 3 Department of Surgery and Medical-Surgical Specialties, Universitat de Barcelona, Barcelona, Spain; 4 IT Department, Hospital Clínic de Barcelona, Barcelona, Spain

## Abstract

This study aims to describe implementing a SNOMED CT-coded health problem (HP) list at Hospital Clínic de Barcelona. The project focuses on enhancing the accuracy and efficiency of clinical coding by automating the process from patient admission, while simultaneously enabling the reuse of coded data for research and management purposes. SNOMED CT was selected as the reference terminology for recording HPs. A subset of terms (our Health Problems Catalogue -HPC-) was created to meet local needs. An NLP tool was integrated into the clinical workstation to assist in primary coding HPs from natural language inputs. The system architecture included four servers (Coder, Reviewer, Manager, and Terminology Server) supporting real-time coding and review processes. Clinical and operational data from April to October 2024 were analyzed to evaluate the system’s performance. Between April 9 and October 4, 2024, a total of 118,534 HPs were recorded. Of these, 74.2% were coded in real-time using the NLP tool, 23.3% were coded by documentation specialists, and 2.5% remained uncoded. The system significantly reduced coding delays and enriched the institutional data warehouse, facilitating real-time research and management activities. Implementing a SNOMED CT-coded HP list supported by NLP and terminology services improved coding accuracy and clinician efficiency. This system enhances clinical understanding, enables evidence-based recommendations, and supports data-driven decision-making in healthcare management and research.

**Clinical Trial Number** Not applicable

## Introduction

### Semantic Interoperability

Semantic interoperability is a fundamental pillar for advanced health information systems, enabling precise and efficient clinical data integration and exchange [1]. This capability is crucial for primary data use in direct patient care and secondary uses such as clinical research, public health management, and data-driven decision-making [2]. Semantic interoperability ensures that clinical concepts and terms are consistently interpreted, reducing variability and improving the quality of care [3].

In this context, the FAIR principles [4] (Findable, Accessible, Interoperable, Reusable) offer a framework for health data govenance, ensuring clinical data is locatable, conditionally accessible, standard-based, and reusable. Adopting terminological standards like SNOMED CT [5] and ICD-10 [6] is fundamental to fulfilling these principles. Despite the decades of availability of these terminologies, many clinical information systems still capture information in plain text or through proprietary code lists [7], hindering the full realization of semantic interoperability.

### Health Problems

A key challenge of semantic interoperability is health problem management. Since Lawrence Weed proposed organizing the Problem-Oriented Medical Record (POMR) in 1968 [8], many attempts have been made to generalize it. However, paper-based records and inconsistent data entry hindered adoption. Since the 1990s, reports in the literature have highlighted the challenges and limitations of implementing a health problem list [9].

With the rise of Electronic Health Records (EHR) [10], the POHR can now serve as the foundation for intelligent health systems, improving clinical understanding and enabling reuse for decision support and research.

### Hospital Clínic de Barcelona (HCB)

In line with the challenges reported in the literature, the management of health problems in the HCB’s information system was, prior to the implementation of the health problem list (HPC) and its integration with SNOMED CT, highly irregular and inconsistent. Entries were not standardized, included free text without coding, and contained inappropriate or ambiguous concepts. This lack of structure made the data neither computable nor retrievable, significantly limiting its utility for clinical decision-making, reporting, or research. Additionally, the problem lists were rarely maintained or updated, leading to redundancy and a lack of relevance over time.

### Objective

The objective of this manuscript is to describe the implementation of a SNOMED CT-coded health problem list at Hospital Clínic de Barcelona. This list utilizes terminology services and a natural language processing (NLP) tool to assist with clinical coding, optimizing the accuracy and efficiency of health problem (HP) coding from the moment of patient admission.

## Methodology

### Terminology Selection and Subset Use

SNOMED CT was selected due to its international recognition for coding natural language expressions, and its ability to link with other classifications and local organizational catalogs [11, 12]. It was chosen over other vocabularies such as ICD for being more comprehensive, clinically focused, and customizable, allowing domain-specific work, different granularity levels, and integration with clinical guidelines and decision support systems.

A comparative study between the Spanish SNOMED extension and the CORE Problem List Subset of SNOMED CT (202111 Version) was performed, covering 160 common hospital discharge diagnoses. The CORE Subset (6,143 concepts) was selected, focusing on the hierarchies: “Finding,” “Disorders,” and “Situation with explicit context.” Additional SNOMED CT codes from primary care referrals, pathology diagnoses, and emergency/outpatient services were added, using ICD-10-CM mappings where necessary.

The Clinical Records Committee at HCB defined the health problem list’s scope, including diagnoses, signs, symptoms, social issues, substance dependencies, and treatment adherence relevant to patient care. A set of General Interest (GI) Health Problems was identified and marked in the Terminology Server (TS) and Hospital Information System (HIS). This HCB is currently not available for download outside HCB.

### Description of the Health Problem List

**Fig. 1:**
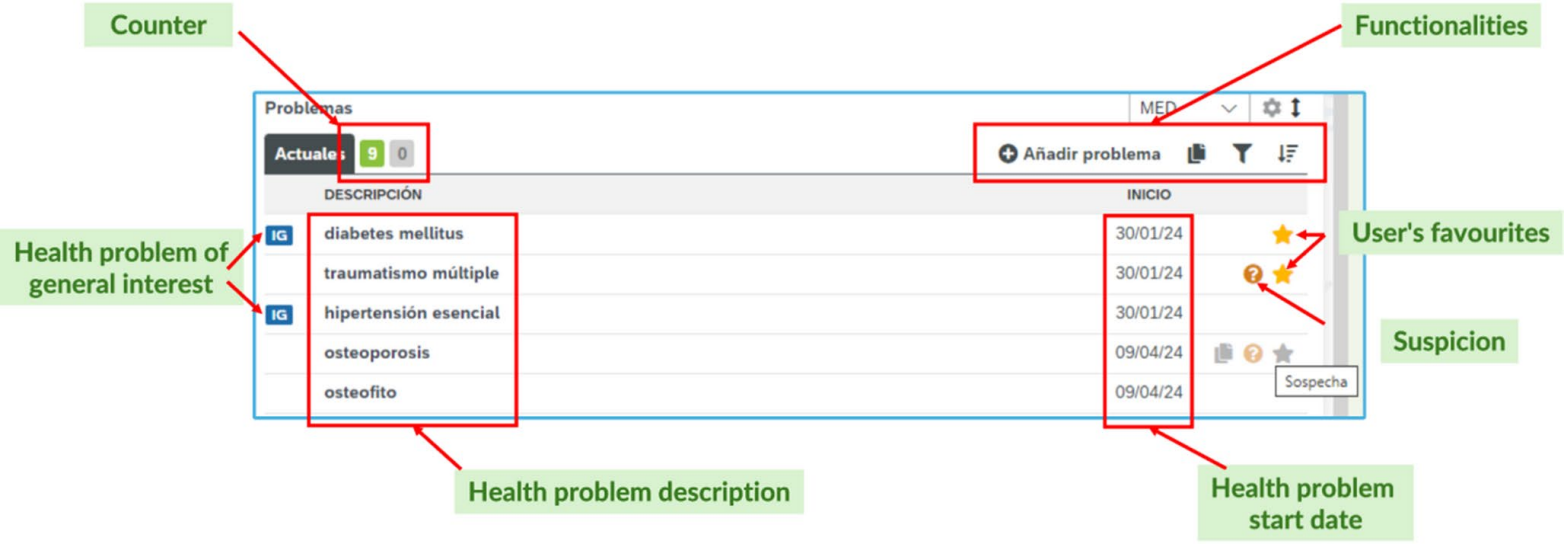
Health problem widget in the clinical workstation

Health problems are entered via an EHR widget specifically designed by HCB’s IT department (Fig. 1), accepting input in Catalan or Spanish.

When a clinician enters data in natural language in the clinical workstation, the text is sent to the coding tool, which returns up to five coded expressions ranked by confidence level. The description displayed in the widget corresponds to the “preferred expression” of SNOMED CT. If the clinician selects “none of the above”, the text is sent for coding by a documentation specialist, and the clinician’s original text is shown in italics until coded.

The main tab differentiates recent active problems from older ones, while deactivated problems appear collapsed separately. Problems are marked as GI, suspected, or favorites, and edits are traceable with auto-filled start and resolved dates. Problems are displayed prioritizing favorites, then GI, then others by recency.

### Architecture Description

**Fig. 2:**
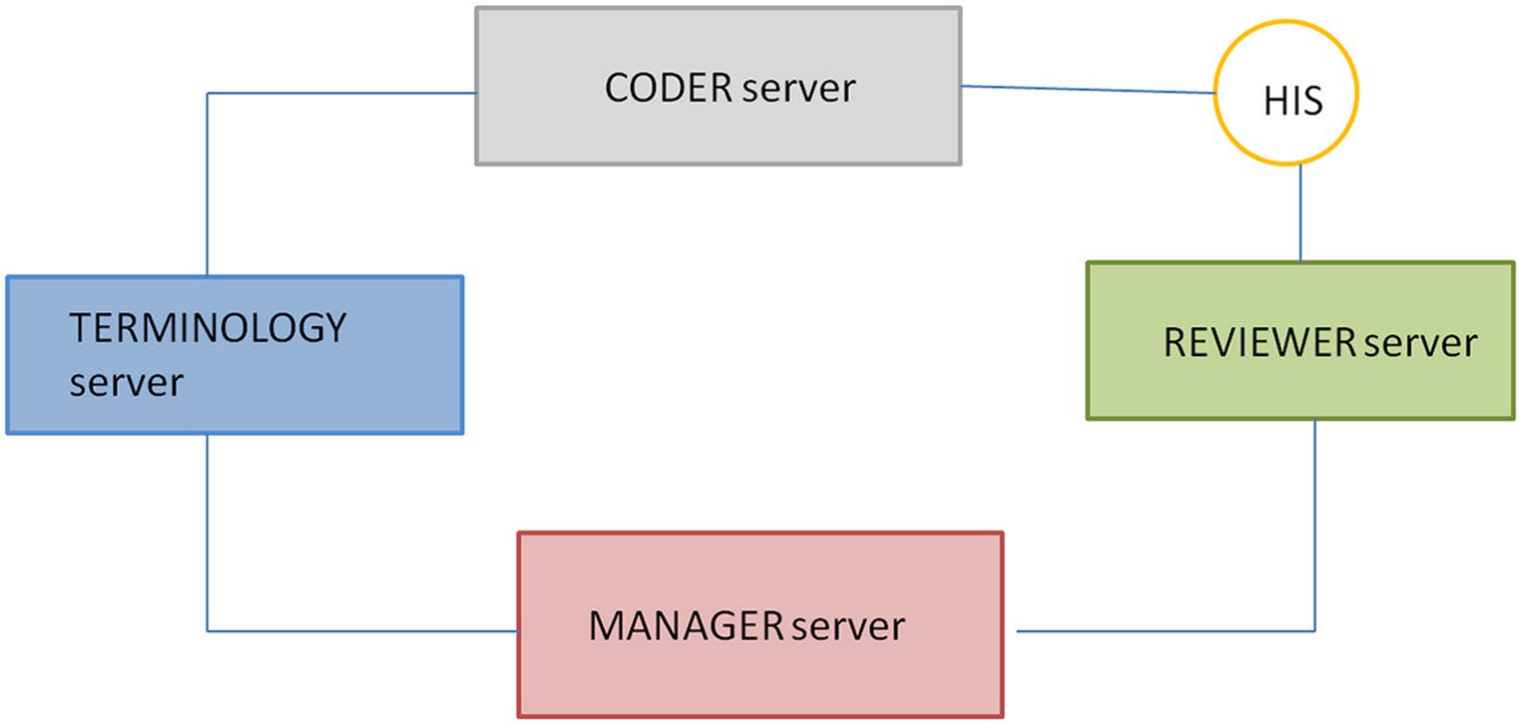
Implemented Architecture

Four virtual external servers—Coder, Reviewer, Manager, and TS—support the infrastructure, separate from the local EHR system (SAP HANA; Fig. 2).

The NLP tool, coding Suite Enterprise (cSE), uses a supervised machine learning system based on a vector classification model (SVM) [13–18]. It correlates data points to a high-dimensional feature space, allowing categorization even when data cannot be linearly separated.

cSE uses morphosyntactic and semantic analysis to group expressions and reduce the set of tests, simplifying classification. Semantic analysis identifies and interprets keywords contextually, considering negations, suspicions, and family history indications, while morphosyntactic analysis focuses on the structure of the sentence. The tool adapts to hospital-specific physician language, and SNOMED CT relationships enhance coding accuracy. cSE was originally developed for ICD-9-CM in the Emergency Department and adapted progressively to ICD-10-CM/PCS, trained on expressions from hospital discharges, outpatient visits, and major surgery reports. Finally, it was further adapted to code health problems using SNOMED CT. For this purpose, the automatic coder was trained using a gold corpus formed from primary care, outpatient, and pathology diagnoses.

The Reviewer platform manages uncoded texts due to low confidence or non-selection, which are submitted regularly.

The Manager handles unresolved issues and updates the HPC, adding new HPs, removing or replacing concepts with more appropriate ones, and managing concept attributes, such as GI. This HPC update process is carried out monthly, in addition to the two annual updates coinciding with the release of the new version of the SNOMED CT terminology. Requests for SNOMED CT additions are submitted when needed to the National Reference Center.

The Terminology Server (TS) synchronizes hospital vocabularies, maintains local subsets, maps concepts between vocabularies and catalogues, and communicates changes automatically to the Manager. It incorporates three SNOMED CT Language Refsets: the International Edition, the International Edition in Spanish, and the Spanish Extension for the National Health System.

The Terminology Services can be accessed directly via its interface or from hospital applications through REST web services. The TS sends automatic notifications to the Manager about health problem catalog concepts affected by updates.

All four servers support texts in three languages (English, Spanish, and Catalan). English serves as the base language, but the servers and clinical workstation communicate in Spanish.

### Implemented Use Cases

In addition to building the HP list that adequately represents the patient’s current clinical scenario, HPs are also linked to clinical actions (CPOE, document signing), with linkage to medication prescription planned for a second phase.

To ensure that HPs represent the patient’s clinical situation appropriately, we implemented a rule that prioritizes active HPs (associated with any action in the last 14 months) and allowed the reconciliation of HPs at discharge.

### Implementation Process of the HP List

Several preparatory actions were carried out for the implementation:

1. Verification of clinical text quality and feasibility of NLP-assisted coding.

A pilot test assessed NLP feasibility on outpatient health problem records, identifying barriers like excessive or insufficient detail, ambiguity, non-HP concepts, multiple problems in one entry, and spelling errors.

2. Analysis of potential sources for retrieving pre-existing health problem information in the HIS.

We identified, coded, and integrated reliable health problem texts from emergency discharge diagnoses, outpatient and pathology diagnoses, primary care referrals, and frequently used departmental diagnoses, ensuring most patients had at least one GI HP recorded.

3. Training the automatic coder with a gold corpus.

The coder was trained on a corpus of primary care, outpatient, and pathology diagnoses. Issues with medical acronyms and abbreviations were addressed by adding 145 identified abbreviations to the corpus.

4. Usability testing of the HP list widget.

Multidisciplinary validation of the widget included scenarios for document creation and test requests, involving physicians from various specialties and Clinical Informatics Service members.

5. Design of the training plan and communication strategy.

A User Manual, Quick Start Guide, and pre-implementation training sessions were developed for clinicians across specialties.

## Results

Table 1 shows the quantitative results of HPs recorded between April 9 and October 4, 2024, while Table 2 displays the results of the clinician selection behavior.

**Table 1:** Quantitative results of HPs

Description	*n*, (%)
Registered HPs	118,534 (100%)
- HPs Coded by physicians using the NLP Tool	87,973 (74.2%)
- HPs Coded by documentation specialists	27,588 (23.3%)
- Uncoded HPs	2,973 (2.5%)

**Table 2:** Clinician selection behavior (*n* = 117,147)

Description	*n*, (%)
Selection of the first candidate concept offered by the NLP tool	63,999 (54.6%)
Selection of one of candidate concepts 2 to 5	29,083 (24.8%)
No selection of suggested candidate concepts (texts sent to documentation specialists)	24,065 (20.6%)

In total, 118,534 health problems (HPs) were recorded in the electronic health record (EHR) between April and October 2024. Of these, 74.2% were coded by practitioners in real time using the NLP tool, 23.3% were manually coded by documentation specialists, and 2.5% remained uncoded (Table 1).

For a subset of 117,147 HPs—those that went through the full coding workflow using the NLP-assisted interface—we analyzed the clinician’s behavior in selecting SNOMED CT suggestions. In 54.6% of cases, the first candidate proposed by the tool was selected. In 24.8%, the clinician chose one of the remaining candidates (positions 2 to 5), while in 20.6% no suggestion was selected, and the case was referred to documentation specialists (Table 2).

The discrepancy between the total number of recorded HPs and those included in the selection analysis is primarily due to transient communication issues between the EHR (SAP) and the coding tool (cSE) at the time of recording, which occasionally prevented the system from logging the clinician’s specific selection action without interrupting the coding workflow nor the data integration. Additionally, a small number of HPs were added retrospectively—for example, validated concepts re-entered directly into the list—or coded manually from free-text sources by documentation staff, bypassing the clinician-facing suggestion interface. These cases are not expected to significantly bias the observed selection patterns.

The implementation of the HP list was generally well-received by clinicians. Only a few user queries were registered regarding importing health problems into discharge summaries. On the other hand, we identified expressions considered to be of low quality (symbols, generic words, ambiguous acronyms, incomplete expressions, etc.), leading to the creation of a list of expressions not accepted in the widget. Entries with these texts were subsequently removed from the HIS and the widget.

Some clinical services requested additions and deletions of concepts from the catalog and requests to create new concepts not present in the terminology. As a result, the HPC was expanded (from 7,364 concepts at implementation to 10,267 at the time of writing), and nine new concepts and/or descriptions were requested from the National Reference Center for SNOMED. The number of GI HPs also increased, from 562 to 896.

## Discussion

### Interoperability and Standardization

This work presents a novel implementation of multi-terminology services at a tertiary academic hospital for standardizing HPs from the start of the care process, supported by an NLP tool that facilitates primary coding. Preliminary results indicate that 74.2% of HPs were recorded in real-time by clinicians, 23.3% by documentation specialists, and only 2.5% remained uncoded.

Accuracy evaluation of auto-coding is inherently limited by minimal or fragmented clinician inputs (e.g., ‘Diab mel 2’ for ‘Type 2 Diabetes Mellitus’), potentially omitting crucial context, which can hinder a comprehensive assessment of coding correctness.

Although the implementation of the health problem list was generally well-received by clinicians, we did not conduct a formal survey to capture qualitative feedback. Future evaluations will include structured surveys or interviews to better understand user experiences, challenges, and areas for improvement in the tool’s usability and adoption.

Having a SNOMED-coded HP list for all patients in hospital, outpatient, and emergency care settings could provide significant benefits to the organization. This implementation allowed us to transition to a structured, coded, and computable health problem list that improves consistency, accuracy, and completeness. The new system created a unique, patient-specific problem list that is maintained and updated regularly, enabling efficient retrieval, easy access, and improved usability for clinicians. Furthermore, our implementation simplifies clinical overviews and facilitates decision-support systems and AI integration. The structured data supports real-time secondary use, enriching institutional research and management activities and avoiding the typical delays of up to a month in obtaining standardized codes after patient discharge.

The integration’s positive reception encourages reuse in other institutional systems (e.g., Pathology, Laboratory Information Systems) through API services, standardizing data recording and usage.

### Comparison with Other Studies

In 1999, Chute et al. [12] published desiderata for clinical terminology services, highlighting the importance of coding a problem list. This seminal article, still relevant today, lists nine key elements. Eight of them (word normalization, word completion, target terminology specification, spell checking, lexical matching, term completion, semantic locality, and term decomposition) have been adequately covered by our implementation. The ninth, term composition (i.e., offering post-coordinated expressions to the user), is planned for the second phase of the project.

Other authors have published their experiences in implementing clinical terminology services. Gambarte et al. [11, 19] created their own SNOMED CT extension with proprietary relationships and defined a permissive term policy that included any term requested by the user, regardless of specificity. Using this approach, 33% of the concepts in their subset could be directly mapped to SNOMED CT concepts. Additionally, 10% of the concepts were deemed invalid and excluded, a figure approximately four times higher than ours.

Silva Layes et al. [20] decided to use graph-oriented databases to implement their terminology services, maximizing the ontological structure of SNOMED CT. This allowed more comprehensive queries and facilitated hierarchy navigation without performance penalties. However, this approach may not be optimal for other terminologies requiring relational persistence.

Metke-Jimenez et al. [7] published their experience with Ontoserver, an FHIR-based clinical terminology service that normalizes communication and facilitates client transitions to other FHIR-based implementations. They used a multi-prefix matching (MPM) algorithm for concept searches, which matches terms whose prefixes align with the user’s input.

Publications on the implementation of health problem lists have existed for over 50 years [21]. Simons et al. [9] conducted a systematic literature review to evaluate factors linked to the success of POMR implementations. One key finding was that an interoperable, multidisciplinary list that provides a time-efficient patient information overview is essential for supporting clinical practice and optimizing care quality.

### Lessons Learned

When implementing a new functionality in an EHR, user-centered design involving resident physicians and other frequent users is critical, given their role in care and documentation.

Additionally, we found that having a dedicated Language Refset to assign preferred descriptions to concepts and use concepts with the necessary granularity for a national and European reference hospital, even if these concepts do not formally exist in the nomenclature, is fundamental.

Planning immediate secondary data use—like integrating health problems into the institutional data warehouse (Datanex) shortly after implementation—demonstrated value to users.

### Limitations

Several limitations should be considered when interpreting this study’s results.

First, since clinician reasoning during coding is inaccessible, we cannot fully verify if the selected SNOMED CT concept matches their intended clinical meaning. This risk may be greater among users with limited documentation habits, potentially introducing inaccuracies, even if such behavior is not expected to be widespread.

Second, the parallel use of ICD-10-CM for discharge diagnoses and SNOMED CT for the health problem list might affect user consistency. ICD-10-CM remains standard for reporting, potentially creating ambiguity or disincentives for clinicians maintaining the problem list. Future developments aim to bridge this gap by mapping ICD-10-CM codes to SNOMED CT concepts and using them to enrich or validate the problem list over time.

Third, the NLP tool’s performance was evaluated in a specific linguistic (Spanish and Catalan) and institutional context. Its generalizability to other settings or languages would require adaptation and retraining.

### Future Directions

The terminology services are integral to the institution’s broader strategy for managing clinical concepts, facilitating data conversion across domains (e.g., pharmacy, clinical records, pathology) for primary and secondary use. Incorporating terminologies (HPO [22], ORPHA [23], ICD-10-CM, and ICD-11, along with plans to include others like LOINC [24]), will enhance scalability. Future efforts may link laboratory and pathology results to HPs, enriching patient data understanding. Work is ongoing to include ORPHA for rare disease coding in the health HP list.

## Conclusion

This manuscript reports the implementation of a SNOMED CT-coded HP list from the outset of the care process using dedicated terminology services. Incorporating a locally validated NLP tool facilitates primary clinical coding, reduces documentation workload, and optimizes accurate concept selection. This initiative marks the first phase of our institutional strategy aimed at standardizing clinical concepts for primary and secondary data use, aligning with FAIR data principles and European initiatives for data interoperability and federated data sharing.

The implementation has encountered no user-related issues, and early data analysis has shown a high percentage of NLP-assisted coding across the institution. The recent inclusion of HP in the data warehouse will motivate users to optimize coding and build a robust and comprehensive HP list.

Concept standardization poses a particular challenge in innovative environments like HCB, where the required granularity and specificity of concepts may not always be reflected in reference terminologies. Change management actions, regular data quality evaluations, and targeted training in departments that require it, along with the adoption of a dedicated Language Refset, are crucial to maintaining sufficient flexibility in concept management.

## Data Availability

The data used in this research can be shared by requesting it from the authors via email.
